# Current News and Opinion

**Published:** 1884-10

**Authors:** 


### Current News and Opinion.

THE TOOTH CROWN PATENTS.

*Editor Independent Practitioner:*

The case of the International Tooth Crown Company vs. Berkowitz & Muller, heard by Judge Wallace in New York last week, was of great importance to the dental profession.

Mr. Dickerson, for the company, moved for an injunction against the defendants under the C. M. Richmond tooth crown patents, the Low bridge patent, and the A. S. Richmond bridge patent.

The motion was opposed by Mr. S. J. Gordon for defendants, the principal witness being Dr. A. S. Richmond, as to the facts concerning the date and use of the alleged inventions, and Drs. Dwinelle, Northrop and Kingsley, as experts upon the date of the art and the scope of the alleged inventions.

The C. M. Richmond and Low patents were invalidated, and the profession has nothing more to fear from them. The decision as to the A. S. Richmond bridge patent was received for consideration.

It may be regarded as open to all to place artificial teeth upon a bridge, supported by *one* Richmond crown or cup. The patent is for such a bridge between *two* Richmond crowns or metal cups, covering roots, at each end of the bridge.

A desperate effort will be made to sustain this patent, which seems to be the only thing of any importance left to the company.

Any dentist in the United States, who placed a bridge sustaining porcelain teeth between two cups or caps covering roots, before

August 8, 1882, can aid materially in defeating that patent by sending his address to Mr. S. J. Gordon, at 44 East 14th St., New York, and stating the time when it was done.

The case is of yet greater importance to parties who have been so unfortunate as to take licenses from the complainant company.

The defendants were licensed, but their licenses had expired before suit was brought, and it was contended that they were estopped, by the stringent and peculiar provisions in the licenses, from showing they were a fraud on the part of the party obtaining them, and therefore void, or that the patents were invalid, because the covenants of the licenses were that they would never at any time deny or contest their validity.

This grave question is also reserved by the court for consideration, but great confidence is felt that the court will never enjoin any parties under patents which it has decided to be invalid, and concerning which the proof was positive that the party obtaining the patents and the licenses knew that the patents were illegal when obtained, and that a great fraud was practiced upon the government and the public by procuring and seeking to enforce them.

W. H. Dwinelle,

Sept. 22, 1884. Pres. Am. Dental Protective Union.

---

### PEDIATRIC APHORISMS.

The *Obstetrical Gazette* quotes the following from Prof. Letamendi (El Dictatem).

1. Children are like the mob; they always complain with reason, although they cannot give the reason why they complain.

2. Always look at the lips of a pale and sickly child; if they are of a deep red color, beware of prescribing tonics internally. At the outset you will congratulate yourself, but in the long run you will repent of having employed them.

3. As a general rule, a sad child has an encephalic lesion, a furious child an abdominal one; a soporific child has both, though indistinctly defined.

4. An attendance on children produces in the mind of an observant physician the conviction that the half, at least, of adult transgressors are so through morbid abdominal influences.

5. A sunny living room, a clean skin, and an ounce of castor-oil in the cupboard—these are the three great points in infantile hygiene.

6. To dispute the clinical value of tracheotomy in croup is a waste of time to no good purpose. Croup or no croup, if there be a positive obstruction to respiration it is but according to reason to open a way for sub-laryngeal respiration. In the day of more knowledge and less nonsense, tracheotomy will be ranked among minor surgical operations.

7. Dentition is a true multiple of pregnancy, in which the uterus and its fœtus become petrified in proportion as they grow. It is not the direct or the eruptive pressure, but the lateral pressure of all together that is the most dangerous. It is from this that so many cerebral symptoms appear, which can in no way be relieved by incision of the gums. The only recourse against the danger of this tranverse pressure is to give the child more nourishment, in the hope that as the general condition is bettered the local condition will also improve.

8. If the incisors of the first dentition are serrated it is bad, but if those of the second formation are the same it is worse. It foretells a number of lesions arising from deficiency of mineral salts in the tissues. There is only one exception, and it is an important one. When the serrated incisors are seen in strong children in whom the fontanelles have closed early, it is a sign of a robust constitution. Instead of a number of small and sharp dentations, there are a few large blunt ones.

9. To regard the eruption of the teeth as the sole factors in the general process known as the first dentition is to perpetrate a sort of a medical synecdoche. Children get their first teeth because they are at the same time getting a second stomach and second intestines.

10. The body of a child possesses such a degree of "acoustic transparency" that in cases of necessity or convenience auscultation may be practiced with the hand, converting it into a telephone, which will reveal as much to the physician as even his ear could do.

11. In practice it is well to distinguish with precision a case in which disease is due to lumbricoids from one in which lumbricoids are due to disease. For in the former case anthelmintics are of service, but in the latter they do harm.

12. Since, until a child is able to speak clearly his relations with the physician are purely objectice, it is very necessary that we should study as carefully as do the veterinarians the exact correspondence between lesions and the expression of the patient.

13. If you wish to cure rapidly and well joint-disease in infants, you must treat them as you would a conflagration—douches, douches, and more douches—until you have succeeded in extinguishing them.

14. The entire system of the moral relation between children and adults should be changed. To speak to them incorrectly merely because they cannot pronounce well; to excite their fears and arouse their weird imaginations simply because they are easily frightened and impressionable; to stimulate their vanity because they are naturally inclined to be vain; these and other similar actions are not only wrong, but absurd.

15. There is, finally, a danger to the women of contracting a vice as yet unregistered in the annals of concupiscence; mastomania, or the sensuality of nursing. When this physiological act degenerates into vice, nursing becomes so frequent as to be nearly continuous, and the result is ruin to both mother and child. Finally, the physician must here, as always, be at once wise, discreet, of good judgment, and firm.

---

### ANALYSES OF BEEF PEPTONOIDS.

Report on Beef Peptonoids by Prof. Attfield, F. R. S., F. I. C., &c., author of "A Manual on Chemistry, General, Medical, and Pharmaceutical."

The chemical examination to which I have submitted your Beef Peptonoids yields the following results in 100 parts:—

| | |
|---|---|
| Albumenoids (containing nitrogen 10.94), | 69.25 |
| Fat, - - - | 10.71 |
| Sugar, including a trace of starch, - | 9.50 |
| Phosphates, equal to bone phosphate, - | 3.01 |
| Other mineral substances, - - | 2.61 |
| Moisture, - - - | 4.92 |
| | 100.00 |

The manufacturers of "Beef Peptonoids" state that this food is composed of dry lean of beef, one-third; the solids of milk, minus most of the fat, one-third; the gluten of wheat, one-third; the beef being partially digested or "peptonized." My analysis fully supports this statement; for I find present between 69 and 70 per cent.

of albumenoids, that is, flesh-forming material (nitrogen 10.94); more than 20 per cent of warmth-producing substance, nearly half of which is milk sugar, and rather more than half fat; 3 per cent. of bone-forming phosphates; about 2 per cent. of other normal mineral matter, and about 5 per cent. of moisture. A sample of the constituent gluten submitted to me was practically pure, containing a mere trace of starch. Rather more than one-fourth of the albumenoids, probably the "peptonized" portion, was soluble; while practically the whole of the "Beef Peptonoids" was readily soluble in peptonizing fluids, showing that it is easily and wholly digested when taken into the stomach. The flavor and odor of the preparation are excellent; its thorough state of dryness fits it for keeping any length of time in any climate. It is by far the most nutritious and concentrated food I have ever met with. Indeed, a palatable and assimilable and in every way acceptable article of food, containing nearly 70 per cent. of truly nutritive nitrogenous material partially peptonized has never before, to my knowledge, been offered to the medical profession or to the public.

JOHN ATTFIELD.

London, November 18, 1883.

---

### DENTISTS' BENEVOLENT ASSOCIATION.

The first annual meeting of the above organization was held at Sweet Springs, Mo., July 10, 1884, the President, Dr. C. H. Darby, in the chair. About twenty-five of its members were present. The report of the Treasurer was received and approved.

The following officers were elected for the ensuing year:

PRESIDENT.

Dr. C. H. Darby, St. Joseph, Missouri.

VICE-PRESIDENTS.

Dr. F. Swan, Booneville, Missouri.
" J. J. R. Patrick, Belleville, Illinois.
" W. H. Eames, St. Louis, Missouri.
" R. E. Nickles, Salina, Kansas.
" J. W. Reed, Butte City, Montana Territory.
Mr. P. X. Combs, Chicago, Illinois.

SECRETARY AND TREASURER.

Dr. R. I. Pearson, Kansas City, Missouri.

BOARD OF DIRECTORS.

Dr. A. H. Thompson, Topeka, Kansas.
" J. A. Price, Weston, Missouri.
" J. M. Austin, St. Joseph, Missouri.
" G. H. Cushing, Chicago, Illinois.
" G. W. Tindall, Kansas City, Missouri.
" C. B. Hewitt, Kansas City, Missouri.
" J. D. Patterson, Kansas City, Missouri.
" H. S. Thompson, Kansas City, Missouri.
" A. C. Schut, Kansas City, Missouri.
" J. S. Letord, Kansas City, Missouri.

This association is duly authorized by charter from the State of Missouri to carry out the objects of its organization, and offers to the members of the dental profession a reliable means of providing for their families in case of death. No salaried officers or other excessive expenses to encumber its progress. Correspondence solicited.
R. I. PEARSON, Secretary,
2206 Troost Ave., Kansas City, Mo.

---

TRANSACTIONS OF THE NEW YORK STATE DENTAL SOCIETY.

Permit me to say to those interested that I have to-day sent by express to the several district secretaries packages of transactions of the Dental Society of the State of New York for 1884, with the request to distribute immediately. Permanent and delegate members will receive their copies through their respective district secretaries. Copies to dental journals, honorary members and non-resident M. D. S's. will be mailed from this place. Yours, etc.,
J. EDW. LINE, Chairman of Publishing Committee.

We have received a copy, and congratulate the committee on its unusually early appearance. It is a matter of regret that " Our Book Table " is crowded out of this number, and with it the proper notice of this volume.—EDITOR.

---

DENTAL EDUCATION IN GERMANY.

Fresh advantages are to be given for the study of dentistry at the University of Berlin. A large building has been given up as the Royal University Clinical Hospital for diseases of the teeth and mouth, under the management of Professor F. Busch, assisted by Dr. Laver and Dr. Klingelhofer. It will be opened next term. A special dental school is also to be opened in the winter term, at the University of Leipzig, under the management of Prof. Dr. Hesse.

NEW ENGLAND DENTAL SOCIETY.

The twenty-second annual meeting of this society will be held at Boston, on Thursday and Friday, Oct. 2 and 3, 1884.

The meeting on Thursday will be at Hawthorn Hall, No. 4 Park St., Boston., and will open at 11 o'clock A. M.

The meeting on Friday will be held at the Old Harvard Medical School Building, foot of North Grove St., Boston. Headquarters will be at the Tremont House.

Members of the profession in New England are cordially invited to attend.

---

DEATH OF COHNHEIM.

This eminent German pathologist, pupil and former assistant of Virchow, Professor of Pathology in the University of Leipzig, died at the latter city August 14th, at the early age of forty-five. His name has long been a familiar one to histologists, owing to his researches on embolism and his discovery of the escape of the white blood-corpuscles from the walls of the vessels during inflammation.

---

FIFTH AND SIXTH DISTRICT SOCIETIES.

The Fifth and Sixth District Dental Societies of the State of New York will hold a Union Semi-Annual Meeting at Syracuse, Tuesday and Wednesday, October 14 and 15, 1884.

The business committee request members to note interesting cases in their office practice in a daily memorandum book, and to favor the meeting with extracts therefrom.

---

ASSISTANT WANTED.

A laboratory assistant is wanted by a dentist doing a first-class operative business, but whose mechanical work is limited. He must be a competent gold worker. Salary moderate. Address "Dental Assistant," in care of the Editor of this Journal.

---

SEVENTH AND EIGHTH DISTRICT DENTAL SOCIETIES.

The above societies will hold a Union Meeting at Rochester the last Tuesday and following Wednesday of this month (Oct. 28th and 29th.) An excellent programme is in preparation.

(26.) In making full sets of teeth I take the bite and arrange the articulation in the usual way and observe all the care of which I am capable, but every little while I am mortified by having the molar teeth too long, so that the front ones will not meet. Cannot some one who has met and conquered this difficulty tell me what on earth is the matter? TROUBLE.

(27.) What is the best thing to use upon the impression before pouring the plaster cast, to prevent its sticking? Oil, soap, collodion, shellac, and other things are used. Is there anything better? I am not quite satisfied with either. D. D. S.

(28.) Where the surfaces of the teeth near the margin of the gums are quite sensitive to the touch (as with the finger nail), what is the best remedy to apply? C. B.

(29.) In cases of extraction, where the blood flows copiously from the socket after the tooth is removed, what is the proper thing to do? L.

(30.) I have been told that no law existed for regulating or restricting the practice of medicine in this State until since the enactment was procured from the Legislature for restricting the practice of dentistry. Is this a fact? NEW YORK.

### Answers.

M. S. (22.) Roughen the surface with a file, and with a rather heavy spatula, heated quite hot, paste or "smear" small pieces of rubber until the surface is covered, taking care to entirely exclude the air. The hot spatula melts the rubber so that it can be easily manipulated, almost like gutta-percha. No dove-tails or drilled holes are required. If a tooth or block is to be put on, fill the space with as much rubber, pasted or smeared in, as seems necessary; then heat the tooth or block carefully, press into place, and when cool trim with the hot spatula.

To invest, use the flask entire (except top plate) fill, bury the plate entirely, put on top plate and bolt loosely if you like. As soon as the plaster sets sufficiently hard to handle, put into the vulcanizer.

This process is not new, but I think is better and quicker than using dissolved rubber, which I used a good deal some years ago, but discarded for what seems to me to be the better way. One or two trials ought to convince the most skeptical. No wax need be used during the entire process. J. B. DAY, N. Y.

M. S. (22.) In repairing, new rubber can be made to cohere perfectly with old rubber, if, just before packing, the old plate be covered with a thin solution of vulcanite dissolved in chloroform. STUDENT.

(24.) Chalfant is still serving his sentence in the California penitentiary. He has about four years more.

(25.) Or write to the undersigned. Richmond obtained, or rather stole what little he knows from Dr. Beers of San Francisco. Beers patented the crown. Richmond added the porcelain tooth front. At present the Beers patent is tied up, some eastern party holding a bill of sale for a deposit upon the proposed purchase money of the patent. Price paid $750.

A. E. BLAKE, D. D. S., San Francisco.

